# Supplementary material for: Modeling glioblastoma heterogeneity as a dynamic network of cell states
Source: Mol Syst Biol. 2021 Sep 16;17(9):e10105. doi: 10.15252/msb.202010105 (PMC8444284; doi:10.15252/msb.202010105)
Supplement: Supplementary file 5 — Source Data for Figure 3 [file MSB-17-e10105-s001.zip › Figure3A_sourcedata/GSEA_3065/hallmarks_state5.GseaPreranked.1623416302988/HALLMARK_MTORC1_SIGNALING.html]

Details for gene set HALLMARK\_MTORC1\_SIGNALING[GSEA]

|  || Dataset | state5 |
| Phenotype | NoPhenotypeAvailable |
| Upregulated in class | na\_neg |
| GeneSet | HALLMARK\_MTORC1\_SIGNALING |
| Enrichment Score (ES) | -0.29359415 |
| Normalized Enrichment Score (NES) | -1.0778494 |
| Nominal p-value | 0.32322055 |
| FDR q-value | 0.61484057 |
| FWER p-Value | 1.0 |
Table: GSEA Results Summary

  

Fig 1: Enrichment plot: HALLMARK\_MTORC1\_SIGNALING      
 Profile of the Running ES Score & Positions of GeneSet Members on the Rank Ordered List

  

| PROBE | GENE SYMBOL | GENE\_TITLE | RANK IN GENE LIST | RANK METRIC SCORE | RUNNING ES | CORE ENRICHMENT || 1 | CD9 |  |  | 14 | 0.857 | 0.0205 | No |
| 2 | HSP90B1 |  |  | 33 | 0.729 | 0.0373 | No |
| 3 | SQLE |  |  | 39 | 0.705 | 0.0549 | No |
| 4 | HSPA5 |  |  | 43 | 0.698 | 0.0726 | No |
| 5 | CANX |  |  | 53 | 0.634 | 0.0879 | No |
| 6 | CALR |  |  | 55 | 0.631 | 0.1041 | No |
| 7 | HMGCR |  |  | 79 | 0.562 | 0.1159 | No |
| 8 | ACSL3 |  |  | 88 | 0.543 | 0.1290 | No |
| 9 | ATP2A2 |  |  | 212 | 0.431 | 0.1262 | No |
| 10 | RDH11 |  |  | 224 | 0.422 | 0.1358 | No |
| 11 | SEC11A |  |  | 247 | 0.411 | 0.1439 | No |
| 12 | SCD |  |  | 284 | 0.391 | 0.1499 | No |
| 13 | CTSC |  |  | 347 | 0.362 | 0.1522 | No |
| 14 | RPN1 |  |  | 351 | 0.360 | 0.1612 | No |
| 15 | TM7SF2 |  |  | 352 | 0.360 | 0.1705 | No |
| 16 | DHCR7 |  |  | 387 | 0.347 | 0.1756 | No |
| 17 | LDLR |  |  | 404 | 0.339 | 0.1825 | No |
| 18 | CXCR4 |  |  | 450 | 0.325 | 0.1858 | No |
| 19 | SERPINH1 |  |  | 460 | 0.322 | 0.1930 | No |
| 20 | STC1 |  |  | 530 | 0.303 | 0.1930 | No |
| 21 | PLOD2 |  |  | 613 | 0.282 | 0.1910 | No |
| 22 | M6PR |  |  | 641 | 0.277 | 0.1951 | No |
| 23 | GLA |  |  | 654 | 0.274 | 0.2008 | No |
| 24 | FKBP2 |  |  | 672 | 0.270 | 0.2058 | No |
| 25 | TFRC |  |  | 727 | 0.255 | 0.2063 | No |
| 26 | FADS2 |  |  | 780 | 0.245 | 0.2067 | No |
| 27 | P4HA1 |  |  | 792 | 0.242 | 0.2117 | No |
| 28 | IDH1 |  |  | 793 | 0.242 | 0.2179 | No |
| 29 | DHCR24 |  |  | 795 | 0.242 | 0.2240 | No |
| 30 | SSR1 |  |  | 922 | 0.221 | 0.2154 | No |
| 31 | TMEM97 |  |  | 995 | 0.211 | 0.2127 | No |
| 32 | EBP |  |  | 1041 | 0.206 | 0.2129 | No |
| 33 | CYP51A1 |  |  | 1061 | 0.203 | 0.2160 | No |
| 34 | SC5D |  |  | 1094 | 0.199 | 0.2175 | No |
| 35 | FADS1 |  |  | 1148 | 0.191 | 0.2164 | No |
| 36 | SLC2A1 |  |  | 1190 | 0.186 | 0.2166 | No |
| 37 | SLC2A3 |  |  | 1209 | 0.183 | 0.2192 | No |
| 38 | ELOVL5 |  |  | 1320 | 0.169 | 0.2111 | No |
| 39 | XBP1 |  |  | 1564 | 0.145 | 0.1873 | No |
| 40 | PIK3R3 |  |  | 1584 | 0.142 | 0.1888 | No |
| 41 | MTHFD2L |  |  | 1597 | 0.141 | 0.1911 | No |
| 42 | ERO1A |  |  | 1804 | 0.127 | 0.1710 | No |
| 43 | HMGCS1 |  |  | 1899 | 0.118 | 0.1633 | No |
| 44 | ELOVL6 |  |  | 1914 | 0.117 | 0.1648 | No |
| 45 | SLC1A4 |  |  | 2049 | 0.108 | 0.1524 | No |
| 46 | IFRD1 |  |  | 2073 | 0.107 | 0.1525 | No |
| 47 | LGMN |  |  | 2091 | 0.106 | 0.1533 | No |
| 48 | BTG2 |  |  | 2174 | 0.100 | 0.1466 | No |
| 49 | ASNS |  |  | 2215 | 0.097 | 0.1446 | No |
| 50 | SDF2L1 |  |  | 2275 | 0.093 | 0.1403 | No |
| 51 | EDEM1 |  |  | 2295 | 0.092 | 0.1405 | No |
| 52 | NUP205 |  |  | 2306 | 0.091 | 0.1417 | No |
| 53 | SLC37A4 |  |  | 2484 | 0.081 | 0.1237 | No |
| 54 | PHGDH |  |  | 2485 | 0.081 | 0.1258 | No |
| 55 | SLC6A6 |  |  | 2565 | 0.076 | 0.1188 | No |
| 56 | SLC7A11 |  |  | 2577 | 0.075 | 0.1194 | No |
| 57 | TBK1 |  |  | 2615 | 0.073 | 0.1171 | No |
| 58 | USO1 |  |  | 2636 | 0.072 | 0.1167 | No |
| 59 | SLC7A5 |  |  | 2658 | 0.071 | 0.1161 | No |
| 60 | CCNF |  |  | 2816 | 0.062 | 0.0999 | No |
| 61 | INSIG1 |  |  | 3055 | 0.049 | 0.0742 | No |
| 62 | TOMM40 |  |  | 3134 | 0.045 | 0.0665 | No |
| 63 | IGFBP5 |  |  | 3230 | 0.040 | 0.0567 | No |
| 64 | ADIPOR2 |  |  | 3460 | 0.029 | 0.0315 | No |
| 65 | PSMC6 |  |  | 3744 | 0.018 | -0.0002 | No |
| 66 | GGA2 |  |  | 3779 | 0.016 | -0.0036 | No |
| 67 | VLDLR |  |  | 3854 | 0.014 | -0.0116 | No |
| 68 | ACACA |  |  | 3928 | 0.010 | -0.0197 | No |
| 69 | IMMT |  |  | 3978 | 0.008 | -0.0250 | No |
| 70 | LTA4H |  |  | 4159 | 0.000 | -0.0454 | No |
| 71 | GBE1 |  |  | 4323 | -0.007 | -0.0638 | No |
| 72 | NFKBIB |  |  | 4459 | -0.013 | -0.0788 | No |
| 73 | MCM4 |  |  | 4534 | -0.015 | -0.0868 | No |
| 74 | GTF2H1 |  |  | 4600 | -0.018 | -0.0937 | No |
| 75 | MCM2 |  |  | 4824 | -0.027 | -0.1183 | No |
| 76 | DDX39A |  |  | 4948 | -0.031 | -0.1314 | No |
| 77 | BUB1 |  |  | 5195 | -0.040 | -0.1583 | No |
| 78 | NUFIP1 |  |  | 5297 | -0.044 | -0.1686 | No |
| 79 | PPP1R15A |  |  | 5321 | -0.045 | -0.1701 | No |
| 80 | DDIT3 |  |  | 5335 | -0.046 | -0.1704 | No |
| 81 | CDC25A |  |  | 5443 | -0.050 | -0.1813 | No |
| 82 | NFYC |  |  | 5574 | -0.056 | -0.1946 | No |
| 83 | CYB5B |  |  | 5644 | -0.060 | -0.2008 | No |
| 84 | PSMD12 |  |  | 5744 | -0.063 | -0.2104 | No |
| 85 | GSR |  |  | 5778 | -0.065 | -0.2125 | No |
| 86 | RIT1 |  |  | 5824 | -0.066 | -0.2159 | No |
| 87 | UFM1 |  |  | 5867 | -0.068 | -0.2189 | No |
| 88 | ALDOA |  |  | 5909 | -0.070 | -0.2218 | No |
| 89 | BHLHE40 |  |  | 5950 | -0.072 | -0.2245 | No |
| 90 | SKAP2 |  |  | 5953 | -0.072 | -0.2228 | No |
| 91 | MAP2K3 |  |  | 5956 | -0.072 | -0.2212 | No |
| 92 | ADD3 |  |  | 5984 | -0.073 | -0.2224 | No |
| 93 | FDXR |  |  | 6009 | -0.074 | -0.2232 | No |
| 94 | UCHL5 |  |  | 6044 | -0.077 | -0.2251 | No |
| 95 | SYTL2 |  |  | 6181 | -0.084 | -0.2383 | No |
| 96 | PSMD14 |  |  | 6275 | -0.089 | -0.2466 | No |
| 97 | SLC9A3R1 |  |  | 6276 | -0.089 | -0.2443 | No |
| 98 | RRM2 |  |  | 6308 | -0.091 | -0.2455 | No |
| 99 | PITPNB |  |  | 6334 | -0.092 | -0.2460 | No |
| 100 | PSME3 |  |  | 6348 | -0.093 | -0.2450 | No |
| 101 | POLR3G |  |  | 6472 | -0.100 | -0.2564 | No |
| 102 | UNG |  |  | 6479 | -0.100 | -0.2545 | No |
| 103 | HMBS |  |  | 6488 | -0.101 | -0.2528 | No |
| 104 | SHMT2 |  |  | 6514 | -0.102 | -0.2530 | No |
| 105 | NMT1 |  |  | 6545 | -0.105 | -0.2538 | No |
| 106 | PLK1 |  |  | 6581 | -0.107 | -0.2550 | No |
| 107 | SRD5A1 |  |  | 6685 | -0.113 | -0.2637 | No |
| 108 | GOT1 |  |  | 6759 | -0.118 | -0.2690 | No |
| 109 | GLRX |  |  | 6822 | -0.123 | -0.2729 | No |
| 110 | COPS5 |  |  | 6916 | -0.130 | -0.2801 | No |
| 111 | GSK3B |  |  | 6951 | -0.133 | -0.2805 | No |
| 112 | DDIT4 |  |  | 6968 | -0.135 | -0.2788 | No |
| 113 | AURKA |  |  | 6990 | -0.136 | -0.2777 | No |
| 114 | PNO1 |  |  | 7028 | -0.139 | -0.2783 | No |
| 115 | CCNG1 |  |  | 7061 | -0.143 | -0.2783 | No |
| 116 | PFKL |  |  | 7197 | -0.155 | -0.2896 | Yes |
| 117 | ACLY |  |  | 7208 | -0.156 | -0.2867 | Yes |
| 118 | EEF1E1 |  |  | 7241 | -0.160 | -0.2862 | Yes |
| 119 | NAMPT |  |  | 7250 | -0.162 | -0.2829 | Yes |
| 120 | PGM1 |  |  | 7268 | -0.164 | -0.2807 | Yes |
| 121 | BCAT1 |  |  | 7285 | -0.166 | -0.2782 | Yes |
| 122 | IDI1 |  |  | 7337 | -0.172 | -0.2795 | Yes |
| 123 | RPA1 |  |  | 7418 | -0.183 | -0.2839 | Yes |
| 124 | HSPA9 |  |  | 7469 | -0.188 | -0.2847 | Yes |
| 125 | GMPS |  |  | 7476 | -0.189 | -0.2805 | Yes |
| 126 | MTHFD2 |  |  | 7486 | -0.190 | -0.2767 | Yes |
| 127 | RRP9 |  |  | 7517 | -0.193 | -0.2751 | Yes |
| 128 | SERP1 |  |  | 7571 | -0.200 | -0.2760 | Yes |
| 129 | ATP6V1D |  |  | 7635 | -0.208 | -0.2777 | Yes |
| 130 | PSMD13 |  |  | 7682 | -0.215 | -0.2774 | Yes |
| 131 | PSAT1 |  |  | 7709 | -0.220 | -0.2747 | Yes |
| 132 | ETF1 |  |  | 7748 | -0.225 | -0.2732 | Yes |
| 133 | TXNRD1 |  |  | 7767 | -0.228 | -0.2694 | Yes |
| 134 | ACTR2 |  |  | 7783 | -0.231 | -0.2652 | Yes |
| 135 | HSPA4 |  |  | 7791 | -0.232 | -0.2600 | Yes |
| 136 | TUBG1 |  |  | 7846 | -0.243 | -0.2598 | Yes |
| 137 | YKT6 |  |  | 7874 | -0.249 | -0.2565 | Yes |
| 138 | DHFR |  |  | 7878 | -0.250 | -0.2504 | Yes |
| 139 | PSMG1 |  |  | 7880 | -0.250 | -0.2440 | Yes |
| 140 | PSMA4 |  |  | 7883 | -0.251 | -0.2378 | Yes |
| 141 | STIP1 |  |  | 7971 | -0.270 | -0.2407 | Yes |
| 142 | HPRT1 |  |  | 7974 | -0.271 | -0.2340 | Yes |
| 143 | PSMA3 |  |  | 8006 | -0.277 | -0.2303 | Yes |
| 144 | ACTR3 |  |  | 8011 | -0.277 | -0.2236 | Yes |
| 145 | GPI |  |  | 8020 | -0.280 | -0.2173 | Yes |
| 146 | PSPH |  |  | 8032 | -0.282 | -0.2113 | Yes |
| 147 | SQSTM1 |  |  | 8044 | -0.284 | -0.2053 | Yes |
| 148 | QDPR |  |  | 8112 | -0.299 | -0.2051 | Yes |
| 149 | PSMC4 |  |  | 8153 | -0.309 | -0.2017 | Yes |
| 150 | UBE2D3 |  |  | 8163 | -0.314 | -0.1946 | Yes |
| 151 | ATP5MC1 |  |  | 8169 | -0.317 | -0.1870 | Yes |
| 152 | PSMB5 |  |  | 8254 | -0.340 | -0.1878 | Yes |
| 153 | LDHA |  |  | 8263 | -0.343 | -0.1799 | Yes |
| 154 | PPA1 |  |  | 8265 | -0.344 | -0.1711 | Yes |
| 155 | G6PD |  |  | 8271 | -0.346 | -0.1628 | Yes |
| 156 | EIF2S2 |  |  | 8314 | -0.358 | -0.1583 | Yes |
| 157 | RAB1A |  |  | 8350 | -0.369 | -0.1527 | Yes |
| 158 | ARPC5L |  |  | 8371 | -0.379 | -0.1452 | Yes |
| 159 | HSPD1 |  |  | 8418 | -0.393 | -0.1403 | Yes |
| 160 | PGK1 |  |  | 8452 | -0.408 | -0.1335 | Yes |
| 161 | CCT6A |  |  | 8469 | -0.418 | -0.1246 | Yes |
| 162 | CACYBP |  |  | 8486 | -0.428 | -0.1153 | Yes |
| 163 | PSMC2 |  |  | 8497 | -0.433 | -0.1053 | Yes |
| 164 | TCEA1 |  |  | 8544 | -0.453 | -0.0989 | Yes |
| 165 | PRDX1 |  |  | 8584 | -0.476 | -0.0910 | Yes |
| 166 | HSPE1 |  |  | 8650 | -0.524 | -0.0849 | Yes |
| 167 | CDKN1A |  |  | 8679 | -0.545 | -0.0740 | Yes |
| 168 | MLLT11 |  |  | 8695 | -0.555 | -0.0614 | Yes |
| 169 | PDAP1 |  |  | 8724 | -0.586 | -0.0495 | Yes |
| 170 | ENO1 |  |  | 8734 | -0.593 | -0.0352 | Yes |
| 171 | PPIA |  |  | 8913 | -0.797 | -0.0348 | Yes |
| 172 | GAPDH |  |  | 8915 | -0.803 | -0.0142 | Yes |
| 173 | TPI1 |  |  | 8939 | -0.852 | 0.0051 | Yes |
Table: GSEA details [plain text format]

  

Fig 2: HALLMARK\_MTORC1\_SIGNALING: Random ES distribution      
 Gene set null distribution of ES for **HALLMARK\_MTORC1\_SIGNALING**
